# Safety and tolerability of first‐line durvalumab with tremelimumab and chemotherapy in esophageal squamous cell carcinoma

**DOI:** 10.1002/cam4.6260

**Published:** 2023-07-25

**Authors:** Dae Ho Lee, Hye Ryun Kim, Bhumsuk Keam, Ken Kato, Yasutoshi Kuboki, Haiyan Gao, Alejandro Yovine, Scott H. Robbins, Myung‐Ju Ahn

**Affiliations:** ^1^ Department of Oncology University of Ulsan College of Medicine, Asan Medical Center Seoul South Korea; ^2^ Division of Medical Oncology, Department of Internal Medicine Yonsei Cancer Center, Yonsei University College of Medicine Seoul South Korea; ^3^ Department of Internal Medicine Seoul National University Hospital Seoul South Korea; ^4^ Department of Head and Neck, Esophageal Oncology National Cancer Center Hospital Tokyo Japan; ^5^ Gastrointestinal Oncology Division National Cancer Center Hospital East Kashiwa Japan; ^6^ AstraZeneca Cambridge UK; ^7^ AstraZeneca Gaithersburg Maryland USA; ^8^ Division of Hematology‐Oncology, Department of Medicine Sungkyunkwan University School of Medicine, Samsung Medical Center Seoul South Korea

**Keywords:** chemotherapy, clinical trials, esophageal squamous cell, immunology

## Abstract

**Background:**

Advanced or metastatic esophageal squamous cell carcinoma (ESCC) is associated with poor prognosis; new first‐line systemic treatment options are needed. Combining immuno‐oncology therapies with standard chemotherapy may represent a promising approach for the treatment of solid tumors. Results from a Phase Ib study evaluating durvalumab with tremelimumab and chemotherapy in patients with advanced or metastatic ESCC are reported.

**Methods:**

Adults with advanced or metastatic ESCC who were candidates for first‐line platinum‐based chemotherapy received durvalumab 1500 mg (Day 1), tremelimumab 75 mg (Day 1), cisplatin 80 mg/m^2^ (Day 1) and 5‐fluorouracil (5‐FU) 800 mg/m^2^ (Days 1–5) in 28‐day cycles until disease progression or discontinuation due to toxicity. The study consisted of safety run‐in (Part A) and expansion (Part B) periods. The primary endpoint was safety. Antitumor activity was an exploratory endpoint.

**Results:**

Sixteen patients were enrolled, 6 in Part A and 10 in Part B, and received a median of 4.0 treatment cycles. All patients were Asian; median age was 65.0 years. All patients experienced adverse events (AEs) related to cisplatin and 5‐FU, and 8 (50.0%) patients experienced AEs related to durvalumab and tremelimumab. Grade ≥3 treatment‐related AEs occurred in 7 (43.8%) patients. There were no deaths associated with AEs. Six (37.5%) patients achieved an objective response. Median progression‐free survival was 3.75 months, and median overall survival was 9.69 months.

**Conclusions:**

Durvalumab with tremelimumab and chemotherapy demonstrated manageable safety and antitumor activity in patients with advanced or metastatic ESCC, warranting further investigation in randomized trials. Registered with ClinicalTrials.gov: NCT02658214.

## 1 INTRODUCTION

Cancers of the esophagus affect a substantial number of individuals worldwide, with an estimated 572,000 new cases and more than 500,000 deaths annually in 2018.^1^ Approximately 65% of patients with esophageal cancers present with locally advanced or metastatic disease, contributing to a poor prognosis and a 5‐year survival rate of 20%.^2^, ^3^ Esophageal squamous cell carcinoma (ESCC) is the predominant type of esophageal cancer and accounts for approximately 90% of cases worldwide, with the highest incidence in eastern Asia.^1^, ^4^ For decades, combination fluoropyrimidine plus platinum‐based chemotherapy (e.g., cisplatin and 5‐fluorouracil [5‐FU]) was recommended as first‐line treatment for patients with advanced or metastatic esophageal cancer.^5^, ^6^ However, minimal improvement in survival has been seen with this combination treatment in patients with esophageal cancer, and new therapeutic strategies are warranted.^7^

Combining cytotoxic chemotherapy with immuno‐oncology therapy, such as immune checkpoint inhibitors (ICIs), can provide synergistic antitumor effects. By inducing tumor cell death, chemotherapy may promote the availability of tumor antigens, which are required to prime the activation of antitumor T cells, and have been associated with the efficacy of ICIs.^8^, ^9^, ^10^ In addition, the elimination of immunosuppressive cells, such as regulatory T cells and myeloid‐derived suppressor cells, has also been observed with chemotherapy and may alter the tumor microenvironment to support the activation of antitumor immune responses with ICIs.^11^, ^12^ Furthermore, chemotherapy has been shown to increase the expression of immunosuppressive molecules targeted by ICIs, including programmed cell death ligand‐1 (PD‐L1).^13^

The benefit of combination treatment with ICIs, or ICIs plus chemotherapy, in patients with untreated, unresectable advanced or metastatic ESCC has been demonstrated in several Phase III clinical trials, including KEYNOTE‐590 (pembrolizumab plus chemotherapy), CheckMate 648 (nivolumab plus chemotherapy and nivolumab plus ipilimumab), and RATIONALE‐306 (tislelizumab plus chemotherapy).^14^, ^15^, ^16^ Based on these findings, combination treatment with ICIs, or ICIs plus chemotherapy, represents a new standard of care for the first‐line treatment of patients with advanced or metastatic ESCC. Pembrolizumab in combination with platinum‐ and fluoropyrimidine‐based chemotherapy is approved for the first‐line treatment of patients with locally advanced or metastatic ESCC in the United States and in Europe, in adults whose tumors express PD‐L1 (combined positive score ≥10).^17^, ^18^ Whereas nivolumab in combination with ipilimumab or platinum‐ and fluoropyrimidine‐based chemotherapy is approved for the first‐line treatment of unresectable advanced or metastatic ESCC in the United States, and in Europe in patients with tumor cell PD‐L1 expression ≥1%.^19^, ^20^

The ICI durvalumab is a selective, high‐affinity IgG1 monoclonal antibody that targets PD‐L1, preventing its interaction with PD‐1.^21^ Durvalumab has demonstrated clinical activity in the treatment of various solid tumor types, including advanced urothelial bladder cancer, non‐small‐cell lung cancer, and head and neck squamous cell carcinoma.^22^, ^23^, ^24^ The ICI tremelimumab is an IgG2 monoclonal antibody that targets cytotoxic T–lymphocyte–associated antigen4 (CTLA‐4).^25^ The combination of an antibody targeting the PD‐1/PD‐L1 pathway with one targeting CTLA‐4 may improve antitumor immune responses via distinct but complementary mechanisms.^26^ As such, dual immuno‐oncology therapy with durvalumab and tremelimumab has been under investigation for the treatment of patients with advanced solid tumors: single tremelimumab regular interval durvalumab (STRIDE) is now approved for unresectable hepatocellular carcinoma globally, including in the United States, Europe, and Japan.^27^, ^28^, ^29^, ^30^, ^31^, ^32^, ^33^, ^34^, ^35^

In this multicohort Phase Ib study (NCT02658214), the safety, tolerability, and exploratory antitumor activity of durvalumab with tremelimumab and chemotherapy were evaluated for the treatment of advanced solid tumors. This study included patients with advanced solid tumors, including patients with head and neck squamous cell carcinoma, small‐cell lung cancer, triple‐negative breast cancer, gastric or gastroesophageal junction cancer, and ESCC. Results from the cohort of patients with ESCC are reported here.

## 2 MATERIALS AND METHODS

### 2.1 Patients

Patients who were eligible for the ESCC cohort included adults aged ≥18 years with histologically or cytologically documented locally advanced unresectable or metastatic ESCC who were candidates for first‐line therapy. Patients with adenosquamous cell carcinoma were eligible for enrollment if squamous cell was the predominant type. Patients who had received neoadjuvant, adjuvant, or definitive chemotherapy or radiotherapy were eligible if the last dose was administered ≥6 months prior to enrollment. Eligible patients had no prior exposure to immune‐mediated therapy, including anti‐PD‐1, anti‐PD‐L1, anti‐PD‐L2, or anti‐CTLA‐4 antibodies, and therapeutic anticancer vaccines. Patients must have had at least one lesion ≥10 mm in diameter with computed tomography or magnetic resonance imaging scans suitable for repeated measurements, per Response Evaluation Criteria in Solid Tumors (RECIST) 1.1 guidelines, an Eastern Cooperative Oncology Group (ECOG) performance status of 0 or 1, and a life expectancy ≥12 weeks. Patients were not eligible if they had active or prior documented autoimmune or inflammatory disorders, history of active primary immunodeficiency, or active infection.

### 2.2 Study design and treatment

This study was a multicenter, open‐label, Phase Ib study that was conducted in four centers in South Korea and two centers in Japan. Informed consent was obtained from patients before any study procedure was conducted. This study was performed in accordance with the Declaration of Helsinki and was consistent with the International Conference on Harmonization Good Clinical Practice Guidelines, applicable regulatory requirements, and the sponsor's policy on Bioethics and Human Biological Samples. The study protocol, its amendments, the informed consent form, and any information provided to the patients were approved by an independent ethical committee or institutional review board at each study center. The approval numbers for each study center are listed in Table S1.

The design of this study was based on nonclinical and clinical studies that have been conducted for tremelimumab, both as a monotherapy and as combination therapy with conventional anticancer agents, to support various cancer indications using different dose schedules.

**FIGURE 1:**
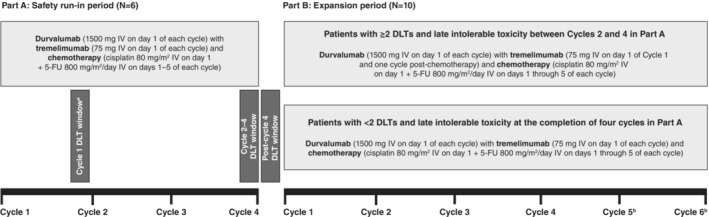
Study design. ^a^: Patients with ≥2 DLTs prior to Cycle 2 were discontinued from the study and did not enter the expansion phase. ^b^:In Part B, patients received 4–6 treatment cycles, as chosen by the investigator. 5‐FU, 5‐fluorouracil; DLT, dose‐limiting toxicity; IV, intravenous.

The study design consisted of a safety run‐in period, Part A, and an expansion period, Part B (Figure 1). In Part A, patients received chemotherapy (cisplatin 80 mg/m^2^ intravenously [IV] on Day 1, with 5‐FU 800 mg/m^2^/day IV administered continuously over 24 h on Days 1 through 5 of each cycle), durvalumab (1500 mg IV administered over 60 min on Day 1 of each cycle), and tremelimumab (75 mg IV administered over 60 min on Day 1 of each cycle) for a total of four 28‐day cycles. Dose‐limiting toxicity (DLT) was evaluated in Part A. If patients experienced ≥2 DLTs before the second treatment cycle in Part A, cohort enrollment was stopped and data were reviewed. If patients experienced ≥2 DLTs between Cycles 2 and 4 in Part A, as well as late intolerable toxicity, they continued into Part B and received standard doses of chemotherapy (as per Part A), durvalumab (as per Part A), and a de‐escalated tremelimumab dose (75 mg IV administered on Day 1 of Cycle 1 and one cycle post‐chemotherapy). If patients experienced <2 DLTs in Part A, as well as late intolerable toxicity after the completion of Cycle 4, they continued into Part B and received the same standard doses of chemotherapy, durvalumab, and tremelimumab administered in Part A. Patients received treatment with chemotherapy, durvalumab, and tremelimumab in Part B for a total of four to six 28‐day treatment cycles, as chosen by the investigator, or until disease progression. Any treatment was discontinued if the patient experienced unacceptable toxicity.

### 2.3 Assessments

The primary endpoint was safety, assessed by incidence of adverse events (AEs) from the time of informed consent until 90 days after the last dose of study treatment. Causality of AEs were assessed by the investigator. AEs of special interest (AESIs) for durvalumab and tremelimumab were monitored and included immune‐mediated AEs, defined as AEs with a potential inflammatory or immune‐mediated mechanism. DLT in Part A was defined as a clinically significant laboratory abnormality (liver transaminase elevation >8 × the upper limit of normal [ULN] or total bilirubin >5 × ULN, Grade 3 or 4 thrombocytopenia with clinically significant bleeding, any Grade 4 thrombocytopenia lasting >48 h, Grade 4 hyponatremia lasting ≥24 h, or alanine aminotransferase or aspartate aminotransferase >5 to 20 × ULN and total bilirubin >1.5 to 3 × ULN), any Grade 4 immune‐related AE not attributable to local tumor response (e.g., inflammatory reaction attributed to local tumor response or inflammatory reaction at sites of metastatic disease or lymph nodes), any Grade ≥3 colitis, any Grade 3 or 4 noninfectious pneumonitis, or any Grade ≥3 AE that did not downgrade to Grade ≤1 or baseline status within 14 days, which were definitively related to the study medication.

Exploratory antitumor activity assessments included objective response rate (ORR; rate of complete response and partial response), duration of response (DoR), disease control rate (DCR; rate of complete response, partial response, and stable disease) at 3 and 12 months, progression‐free survival (PFS) and overall survival (OS). Tumor response was evaluated according to RECIST 1.1 and confirmed by the investigator.

### 2.4 Statistical analyses

The safety analysis was performed in all patients who received ≥1 dose of study treatment. The efficacy analysis was performed in the response evaluable set, which included all patients who received ≥1 dose of study treatment who had a tumor assessment and measurable disease at baseline.

Data are summarized with descriptive statistics. Continuous variables were summarized by the number of observations and median with upper and lower quartiles, range or 95% confidence interval (CI). Categorical values were summarized by frequency and percentage. Kaplan–Meier methods were used to estimate the median, 95% CIs, and upper and lower quartile ranges for DoR, PFS, and OS. No inferential analyses were performed based on statistical tests. Data are presented for Part A and Part B separately and in combination.

## 3 RESULTS

### 3.1 Patient disposition

The data cutoff for this analysis was November 11, 2019.

Six patients were enrolled into Part A between November 13, 2017 and February 14, 2018. All patients in Part A received ≥1 dose of durvalumab and tremelimumab; no patients experienced DLTs. In Part A, reasons for discontinuation included disease progression (durvalumab, *n* = 6; tremelimumab, *n* = 2) and maximum number of cycles of immunotherapy reached (tremelimumab, *n* = 4).

Ten patients were enrolled into Part B between July 26, 2018 and January 11, 2019. As no DLTs were experienced in Part A, no dose modifications were required in Part B, and all patients received ≥1 standard dose of chemotherapy, durvalumab, and tremelimumab. In Part B, reasons for discontinuation of durvalumab included patient decision (*n* = 1), occurrence of AEs (*n* = 3), or disease progression (*n* = 6), and reasons for discontinuation of tremelimumab included patient decision (*n* = 1), occurrence of AEs (*n* = 2), disease progression (*n* = 1), or maximum number of cycles of immunotherapy reached (*n* = 6).

### 3.2 Demographics and baseline characteristics

All 16 patients were Asian, the median age of the total cohort was 65.0 years, and most patients were male (81.3%; Table 1). Of 11 patients with smoking status data available, 4 patients were current smokers and 7 were former smokers. Most patients had an ECOG performance status of 1 (75%) and extent of disease that was classed as both locally advanced and metastatic (87.5%). There were seven (43.8%) patients who had received previous neoadjuvant, adjuvant, or definitive chemotherapy or radiotherapy: five (31.3%) patients had received previous radiotherapy, seven (43.8%) patients had received previous chemotherapy (cisplatin and 5‐FU); all seven (43.8%) patients had received ≥2 previous treatment regimens, including radiotherapy and cisplatin and 5‐FU. Demographics and baseline characteristics were similar across Part A and Part B (Table 1).

**TABLE 1:** Patient demographics and baseline characteristics.

Parameter	Part A (*N* = 6)	Part B (*N* = 10)	Total (*N* = 16)
Age, median (range), year	56.0 (44–77)	65.5 (37–68)	65.0 (37–77)
Sex, male, *n* (%)	5 (83.3)	8 (80.0)	13 (81.3)
Race, Asian, *n* (%)	6 (100)	10 (100)	16 (100)
Smoking status			
Current smoker	1 (16.7)	3 (30.0)	4 (25.0)
Former smoker	2 (33.3)	5 (50.0)	7 (43.8)
Not available	3 (50.0)	2 (20.0)	5 (31.3)
ECOG performance status, *n* (%)			
0	0	4 (40.0)	4 (25.0)
1	6 (100)	6 (60.0)	12 (75.0)
Extent of disease, *n* (%)			
Metastatic	1 (16.7)	1 (10.0)	2 (12.5)
Locally advanced	0	0	0
Both	5 (83.3)	9 (90.0)	14 (87.5)
Previous treatment, *n* (%)			
Any	4 (66.7)	3 (30.0)	7 (43.8)
Radiotherapy	3 (50.0)	2 (20.0)	5 (31.3)
Cytotoxic chemotherapy	4 (66.7)	3 (30.0)	7 (43.8)
Number of previous treatment regimens, *n* (%)			
0	2 (33.3)	7 (70.0)	9 (56.3)
1	0	0	0
2	4 (66.7)	2 (20.0)	6 (37.5)
3	0	1 (10.0)	1 (6.3)

Abbreviations: ECOG, Eastern Cooperative Oncology Group.

### 3.3 Safety

In Part A and Part B, the median number of cycles of treatment with cisplatin, 5‐FU, durvalumab, and tremelimumab completed was 4.0. The median (range) total treatment durations were 105.0 (24–161) days for cisplatin, 106.5 (24–166) days for 5‐FU, 123.5 (38–293) days for durvalumab, and 112.0 (38–129) days for tremelimumab.

All patients experienced ≥1 AE (Table 2). AEs causally related to durvalumab or tremelimumab occurred in eight (50.0%) patients each, and all patients experienced ≥1 AE causally related to cisplatin and 5‐FU. Grade ≥3 AEs that were causally related to any treatment occurred in seven (43.8%) patients and included decreased neutrophil count (25.0%), anemia (6.3%), abnormal hepatic function (6.3%), myositis (6.3%), increased alanine aminotransferase (6.3%), increased amylase (6.3%), and increased aspartate aminotransferase (6.3%). Serious AEs were reported in seven (43.8%) patients: serious AEs of pneumonia (12.5%), monoplegia (6.3%), syncope (6.3%), pyrexia (6.3%), cervical vertebral fracture (6.3%), and infusion‐related reaction (6.3%) were not considered to be related to study treatment. Four (25.0%) patients had serious AEs causally related to a study treatment, which included dehydration (6.3%), abnormal hepatic function (6.3%), myositis (6.3%), and decreased neutrophil count (6.3%). There were three (18.8%) patients with ≥1 AE leading to discontinuation of any study treatment; these AEs included monoplegia, increased alanine aminotransferase, increased aspartate aminotransferase, cervical vertebral fracture, and infusion‐related reaction. No patient experienced an AE with an outcome of death. The most common AEs overall are provided in Table S2, and included nausea (75%), constipation (43.8%), and decreased neutrophil count (43.8%).

**TABLE 2:** Summary of AEs in the ESCC Cohort.

AE, *n* (%)	Part A (*N* = 6)	Part B (*N* = 10)	Total (*N* = 16)
Any AE	6 (100)	10 (100)	16 (100)
AE related to any treatment^a^	6 (100)	10 (100)	16 (100)
Durvalumab‐related^a^	2 (33.3)	6 (60.0)	8 (50.0)
Tremelimumab‐related^a^	2 (33.3)	6 (60.0)	8 (50.0)
5‐FU‐related^a^	6 (100)	10 (100)	16 (100)
Cisplatin‐related^a^	6 (100)	10 (100)	16 (100)
Grade ≥3 AE	5 (83.3)	5 (50.0)	10 (62.5)
Treatment‐related Grade ≥3 AE^a^	3 (50.0)	4 (40.0)	7 (43.8)
Serious AE	3 (50.0)	4 (40.0)	7 (43.8)
Treatment‐related serious AE^a^	1 (16.7)	3 (30.0)	4 (25.0)
AE leading to discontinuation of any treatment	0	3 (30.0)	3 (18.8)
Leading to discontinuation of durvalumab	0	3 (30.0)	3 (18.8)
Leading to discontinuation of tremelimumab	0	2 (20.0)	2 (12.5)
Leading to discontinuation of 5‐FU	0	3 (30.0)	3 (18.8)
Leading to discontinuation of cisplatin	0	3 (30.0)	3 (18.8)
AE with outcome of death	0	0	0

Abbreviations: 5‐FU, 5‐fluorouracil; AE, adverse event; ESCC, esophageal squamous cell carcinoma.

^a^ As assessed by the investigator.

Seven (43.8%) patients experienced ≥1 AESI (Table S3). These included diarrhea (18.8%), infusion‐related reaction (12.5%), rash (6.3%), enterocolitis (6.3%), hyperthyroidism (6.3%), and myositis (6.3%). One patient experienced an infusion‐related reaction that led to treatment discontinuation, and one patient experienced an AESI requiring steroids (myositis), both occurring in Part B. Immune‐mediated AEs, as reported by investigators, were reported in eight (50.0%) patients. Of the reported immune‐mediated AEs, AEs of pruritis (18.8%), hyperthyroidism (6.3%), diarrhea (6.3%), rash (6.3%), and myositis (6.3%) met the criteria for immune‐mediated AEs, which were prespecified in the study protocol.

### 3.4 Antitumor activity

**FIGURE 2:**
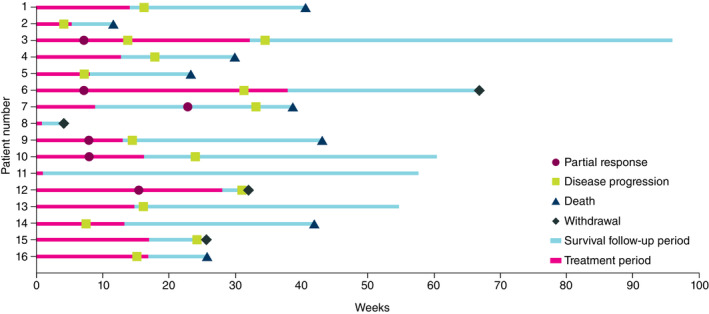
Clinical responses and follow‐up period in the ESCC cohort (Part A: Patients 1–6; Part B: Patients 7–16). ESCC, esophageal squamous cell carcinoma.

Confirmed objective responses were observed in six patients (ORR, 37.5%; 95% CI, 15.20–64.57); all were partial responses (Table 3). The median DoR was 3.0 months (Q1, 1.9; Q3, 3.7). DCRs were 68.8% and 37.5% at 3 and 12 months, respectively. Median (95% CI) PFS and OS were 3.75 (1.71–6.87) and 9.69 (5.95 to not calculable) months, respectively. Clinical responses for individual patients are presented in Figure 2. The percent changes in tumor size (Figure S1) and representative scans from patients who achieved a response (Figure S2) are included in the supporting material.

**TABLE 3:** Summary of antitumor activity.

Assessment	Part A (*N* = 6)	Part B (*N* = 10)	Total (*N* = 16)
Objective response rate, *n* (% [95% CI])	2 (33.3 [4.33–77.72])	4 (40.0 [12.16–73.76])	6 (37.5 [15.20–64.57])
Best overall response, *n* (%)			
Complete response	0	0	0
Partial response	2 (33.3)	4 (40.0)	6 (37.5)
Stable disease	2 (33.3)	3 (30.0)	5 (31.3)
Disease progression^a^	2 (33.3)	1 (10.0)	3 (18.8)
Median DoR, months (Q1, Q3)	0	3.0 (1.9, 3.7)	3.0 (1.9, 3.7)
Disease control rate, *n* (%)			
3 months	4 (66.7)	7 (70.0)	11 (68.8)
12 months	2 (33.3)	4 (40.0)	6 (37.5)
Median PFS, months (95% CI)	3.75 (0.95–NC)	4.62 (1.71–7.16)	3.75 (1.71–6.87)
Median OS, months (95% CI)	8.13 (2.73–NC)	9.95 (5.95–NC)	9.69 (5.95–NC)

Abbreviations: CI, confidence interval; DoR, duration of response; NC, not calculable; OS, overall survival; PFS, progression‐free survival; Q1, lower quartile; Q3, upper quartile; RECIST, Response Evaluation Criteria in Solid Tumors.

^a^ Includes patients without complete or partial response (according to RECIST 1.1 or unconfirmed), those without stable disease and those who died.

## 4 DISCUSSION

Results from the ESCC cohort of this Phase Ib study support a manageable safety profile of durvalumab with tremelimumab and chemotherapy for treatment of patients with advanced or metastatic ESCC. No unexpected safety events occurred that could be causally associated to study treatment by the investigators. In addition, most treatment‐related AEs that occurred were related to chemotherapy. The safety profile for durvalumab with tremelimumab and chemotherapy was generally consistent with other ICI studies in untreated advanced or metastatic ESCC, including KEYNOTE‐590 and CheckMate 648.^14^, ^15^

Results from exploratory assessments in this study demonstrated antitumor activity with durvalumab in combination with tremelimumab and chemotherapy in patients with advanced or metastatic ESCC. In this study, the ORR was 37.5% and median DoR was 3.0 months. In addition, median OS and PFS were 9.7 and 3.8 months, respectively.

The ORR and median OS reported in this study were generally comparable to prospective and retrospective studies of chemotherapy alone for the treatment of patients with advanced or metastatic ESCC, which have reported ORRs, ranging from 33.3% to 37.2%, and OS, ranging from 7 to 10 months.^7^, ^36^, ^37^, ^38^

When compared with Phase III trials of ICIs combined with chemotherapy, including KEYNOTE‐590, CheckMate 648, and RATIONALE‐306, ORRs were generally lower, and median DoR, OS, and PFS were generally shorter in this study.^14^, ^15^, ^16^ ORRs reported for pembrolizumab, nivolumab, or tislelizumab in combination with chemotherapy ranged from 45.0% to 63.0%, whereas median DoR ranged from 7.1 to 8.4 months, and median OS and PFS ranged from 12.4 to 17.2 months and 5.8 to 7.3 months, respectively.^14^, ^15^, ^16^

Several reasons may explain the lower clinical response in this study compared with KEYNOTE‐590, CheckMate 648, and RATIONALE‐306, including the small number of patients enrolled in this study and the number of treatment cycles received.^14^, ^15^, ^16^ Further randomized trials with larger patient populations are needed to fully assess the antitumor activity of this regimen and to compare directly with current standards of care.

Limitations of the current study include the small number of patients enrolled in the ESCC cohort and the uncontrolled, single‐arm design. Overall, durvalumab with tremelimumab and chemotherapy demonstrated manageable safety and antitumor activity in patients with advanced or metastatic ESCC, warranting further investigation in randomized clinical trials.

## AUTHOR CONTRIBUTIONS

**Dae Ho Lee:** Data curation (equal); formal analysis (equal); supervision (equal); visualization (equal); writing – review and editing (equal). **Hye Ryun Kim:** Data curation (equal); formal analysis (equal); visualization (equal); writing – review and editing (equal). **Bhumsuk Keam:** Data curation (equal); formal analysis (equal); visualization (equal); writing – review and editing (equal). **Ken Kato:** Data curation (equal); formal analysis (equal); visualization (equal); writing – review and editing (equal). **Yasutoshi Kuboki:** Data curation (equal); formal analysis (equal); supervision (equal); visualization (equal); writing – review and editing (equal). **Haiyan Gao:** Data curation (equal); formal analysis (equal); supervision (equal); visualization (equal); writing – review and editing (equal). **Alejandro Yovine:** Conceptualization; supervision (equal); writing – review and editing (equal). **Scott H. Robbins:** Data curation (equal); formal analysis (equal); supervision (equal); visualization (equal); writing – review and editing (equal). **Myung‐Ju Ahn:** Data curation (equal); formal analysis (equal); supervision (equal); visualization (equal); writing – review and editing (equal).

## FUNDING INFORMATION

This study was funded by AstraZeneca.

## CONFLICT OF INTEREST STATEMENT

Dae Ho Lee: Personal fees: AbbVie, AstraZeneca, BC Pharma, Boehringer Ingelheim, Bristol‐Myers Squibb, ChongKeunDang, CJ Healthcare, Eli Lilly, Genexine, Janssen, Menarini, Merck, MSD, Mundipharma, Novartis, ONO, Pfizer, Roche, Samyang Biopharm, ST Cube, Takeda. Non‐financial support: Blueprint Medicines, Takeda. Hye Ryun Kim: Consultant/advisor: AstraZeneca, Boehringer Ingelheim, Bristol‐Myers Squibb, MSD, Takeda. Honoraria: AstraZeneca, Boehringer Ingelheim, Bristol‐Myers Squibb, MSD, ONO. Research funding: AstraZeneca, Bristol‐Myers Squibb, ONO, YUHAN. Bhumsuk Keam: Research funding: AstraZeneca, MSD Oncology, ONO. Consultant/advisor: ABL Bio, AstraZeneca, CbsBioscience, Cellid, Genexine, Handok, MSD Oncology. Honoraria: AstraZeneca, Merck, MSD Oncology. Ken Kato: Research funding: AstraZeneca, Bayer, BeiGene, Chugai, Merck, Merck Serono, MSD, Oncolys BioPharma, ONO, Shionogi. Yasutoshi Kuboki: Research funding: Amgen, Astellas, AstraZeneca, Boehringer Ingelheim, Chugai, Daiichi‐Sankyo, Genmab, GSK, Incyte, Lilly, ONO, Taiho, Takeda. Honoraria: Bayer, Bristol‐Myers Squibb, Lilly, Merck Serono, ONO, Taiho. Haiyan Gao, Alejandro Yovine, and Scott H. Robbins: Employees/shareholders: AstraZeneca. Myung‐Ju Ahn: Consultant/advisor: Alpha Pharmaceutical, AstraZeneca, Bristol‐Myers Squibb, MSD, Novartis, Roche, Takeda. Honoraria: AstraZeneca, Bristol‐Myers Squibb, MSD, ONO, Roche.

## ETHICS STATEMENT

This study was performed in accordance with the Declaration of Helsinki and was consistent with the International Conference on Harmonization Good Clinical Practice Guidelines, applicable regulatory requirements and the sponsor's policy on Bioethics and Human Biological Samples. The study protocol, its amendments, the informed consent form, and any information provided to the patients were approved by an independent ethical committee or institutional review board at each study center. The approval numbers for each study center are listed in Table S1.

## INFORMED CONSENT

Informed consent was obtained from patients before any study procedure was conducted.

## CLINICAL TRIAL REGISTRATION

Registered with ClinicalTrials.gov: NCT02658214.

## PRÉCIS

In this Phase Ib study, durvalumab and tremelimumab in combination with first‐line chemotherapy demonstrated manageable safety and antitumor activity in patients with advanced or metastatic esophageal squamous cell carcinoma.

## Supporting information


Data S1:

## Data Availability

Data underlying the findings described in this manuscript may be obtained in accordance with AstraZeneca's data sharing policy described at: https://astrazenecagrouptrials.pharmacm.com/ST/Submission/Disclosure. Data for studies directly listed on Vivli can be requested through Vivli at www.vivli.org. Data for studies not listed on Vivli can be requested through Vivli at https://vivli.org/members/enquiries‐about‐studies‐not‐listed‐on‐the‐vivli‐platform/. AstraZeneca Vivli member page is also available outlining further details: https://vivli.org/ourmember/astrazeneca/.
