# Supplementary material for: Safety and tolerability of first‐line durvalumab with tremelimumab and chemotherapy in esophageal squamous cell carcinoma
Source: Cancer Med. 2023 Jul 25;12(15):16066–75. doi: 10.1002/cam4.6260 (PMC10469840; doi:10.1002/cam4.6260)
Supplement: Supplementary file 1 — Data S1: [file CAM4-12-16066-s001.docx]

**SUPPORTING INFORMATION**

**Table S1.** List of IRBs, and Approval Numbers, for Each Study Center

| Name of IRB | Approval Number |
| --- | --- |
| Asan Medical Center Institution Review Board | 2016-0313 |
| Severance IRB | 4-2016-0102 |
| IRB of Seoul National University Hospital | 1603-045-747 |
| National Cancer Ctr IRB#2-j | T4474 |
| National Cancer Ctr IRB#2-j | K0660 |
| Samsung Medical Center (SMC) IRB (Institutional Review Board) | 2016-02-141 |

Abbreviation: IRB, institutional review board.

**Table S2.** Most Common AEs (≥15%)

| AE, n (%) | Part A (N=6) | Part B (N=10) | Total (N=16) |
| --- | --- | --- | --- |
| Nausea | 5 (83.3) | 7 (70.0) | 12 (75.0) |
| Neutrophil count decreased | 4 (66.7) | 3 (30.0) | 7 (43.8) |
| Constipation | 1 (16.7) | 6 (60.0) | 7 (43.8) |
| Decreased appetite | 2 (33.3) | 4 (40.0) | 6 (37.5) |
| Stomatitis | 3 (50.0) | 3 (30.0) | 6 (37.5) |
| Alanine aminotransferase increased | 1 (16.7) | 4 (40.0) | 5 (31.3) |
| Aspartate aminotransferase increased | 1 (16.7) | 4 (40.0) | 5 (31.3) |
| Dyspepsia | 3 (50.0) | 2 (20.0) | 5 (31.3) |
| Cough | 3 (50.0) | 1 (10.0) | 4 (25.0) |
| Platelet count decreased | 2 (33.3) | 2 (20.0) | 4 (25.0) |
| Weight decreased | 1 (16.7) | 3 (30.0) | 4 (25.0) |
| Diarrhea | 3 (50.0) | 0 | 3 (18.8) |
| Pruritis | 1 (16.7) | 2 (20.0) | 3 (18.8) |
| Pyrexia | 1 (16.7) | 2 (20.0) | 3 (18.8) |
| Vomiting | 1 (16.7) | 2 (20.0) | 3 (18.8) |
| Dizziness | 2 (33.3) | 1 (10.0) | 3 (18.8) |
| Productive cough | 1 (16.7) | 2 (20.0) | 3 (18.8) |
| Hiccups | 1 (16.7) | 2 (20.0) | 3 (18.8) |
| Skin hyperpigmentation | 3 (50.0) | 0 | 3 (18.8) |

Abbreviations: AE, adverse event.

**Table S3.** All AESIs, Including Immune-mediated (Investigator-assessed) AEs

| AE, n (%) | Part A (N=6) | Part B (N=10) | Total (N=16) |
| --- | --- | --- | --- |
| Any AESI | 4 (66.7) | 3 (30.0) | 7 (43.8) |
| Diarrhea | 3 (50.0) | 0 | 3 (18.8) |
| Infusion-related reaction | 1 (16.7) | 1 (10.0) | 2 (12.5) |
| Hyperthyroidism | 1 (16.7) | 0 | 1 (6.3) |
| Rash | 1 (16.7) | 0 | 1 (6.3) |
| Enterocolitis | 0 | 1 (10.0) | 1 (6.3) |
| Myositis | 0 | 1 (10.0) | 1 (6.3) |
| Any immune-mediated AE^a^ | 2 (33.3) | 6 (60.0) | 8 (50.0) |
| Pruritis | 1 (16.7) | 2 (20.0) | 3 (18.8) |
| Alanine aminotransferase increased^b^ | 0 | 3 (30.0) | 3 (18.8) |
| Aspartate aminotransferase increased^b^ | 0 | 3 (30.0) | 3 (18.8) |
| Hyperthyroidism | 1 (16.7) | 0 | 1 (6.3) |
| Diarrhea | 1 (16.7) | 0 | 1 (6.3) |
| Rash | 1 (16.7) | 0 | 1 (6.3) |
| Amylase increased^b^ | 0 | 1 (10.0) | 1 (6.3) |
| Anemia^b^ | 0 | 1 (10.0) | 1 (6.3) |
| Dehydration^b^ | 0 | 1 (10.0) | 1 (6.3) |
| Constipation^b^ | 0 | 1 (10.0) | 1 (6.3) |
| Enterocolitis | 0 | 1 (10.0) | 1 (6.3) |
| Hepatic function abnormal^b^ | 0 | 1 (10.0) | 1 (6.3) |
| Dry skin^b^ | 0 | 1 (10.0) | 1 (6.3) |
| Myositis | 0 | 1 (10.0) | 1 (6.3) |
| Fatigue^b^ | 0 | 1 (10.0) | 1 (6.3) |

Abbreviations: AE, adverse event; AESI, adverse event of special interest.

^a^As assessed by the investigator, using criteria that may or may not be specifically defined in the study protocol.

^b^AE not considered to be immune-mediated according to criteria defined in the study protocol.

**Figure S1.** Percent change from baseline in tumor size

**Figure S2.** (**A**) Representative patient orbital MRI (top) and chest CT (bottom) scans at baseline and week 7: right orbital metastasis decreased from 14 mm at baseline to 0 mm at week 7 (complete response). Esophageal primary tumor decreased in size from baseline to week 7, with no change in mediastinal lymph nodes. Overall response: partial response. (**B**) Representative patient abdominal CT scans at baseline and week 7: left paraaortic lymph node metastasis decreased in size from 15 mm at baseline to 10 mm at week 7, with decreased size of multiple retroperitoneal metastatic lymph nodes <5 mm and a scanty amount of pelvic ascites. Overall response: partial response

CT, computed tomography; MRI, magnetic resonance imaging
